# How much Copper Do Calves Need? Retention and Hepatic Storage in Artificially Reared Milk-Fed Holstein–Friesian Calves

**DOI:** 10.1007/s12011-026-05190-7

**Published:** 2026-07-01

**Authors:** Amy P. Marsh, Liam A. Sinclair, Joe M. Roberts, Alexander M. Mackenzie, Stefan E. Yerby, James A. Huntington, James H. McCaughern

**Affiliations:** 1 Animal Science Research Centre, Harper Adams University, Newport, Shropshire UK; 2 Agriculture and Environment Department, Harper Adams University, Newport, Shropshire, UK; 3 College of Medical, Veterinary & Life Sciences, University of Glasgow, Glasgow, UK

**Keywords:** Hepatic retention, Calves, Liver copper, Toxicity, Weaning

## Abstract

Excessive copper (Cu) absorption in calves may increase the risk of excessive hepatic Cu accumulation, likely driven by their elevated pre-ruminant absorptive capacity, with the potential for harmful Cu accumulation leading to adverse health and productivity outcomes. However, quantitative data across the weaning transition remains limited. This study utilized secondary data and bio-banked samples from 35 Holstein–Friesian bull calves to estimate hepatic Cu retention coefficients pre- and post-weaning. Although mean hepatic Cu concentrations remained broadly stable throughout the study, between 311 and 675 mg/kg of DM, changes varied by developmental stage: calves experienced hepatic Cu accumulation of 2.5 mg of Cu/kg of DM/d pre-weaning but a net reduction of 1.8 mg/kg of DM/d post-weaning. Pre-weaning calves exhibited a hepatic Cu retention coefficient of 81% compared to post-weaning calves, where the hepatic Cu retention coefficient was 5%. This reduction in absorptive capacity is consistent with increased rumen development and greater antagonism from dietary sulfur and molybdenum. Hepatic steady-state Cu intake requirements were estimated at 3 mg/kg of DM pre-weaning and 15 mg/kg of DM post-weaning. These findings demonstrate that the pre-weaning period represents a phase of high Cu retention and heightened susceptibility to hepatic Cu accumulation, even when dietary Cu meets current recommendations. Refining Cu feeding guidelines to more accurately reflect developmental stage and rumen maturation may help prevent excessive hepatic Cu loading while maintaining adequate mineral supply.

## Introduction

Copper (Cu) is an essential trace element required by all living organisms [1]. In mammals, it plays a critical role in the activity of metalloproteins and functions as a cofactor for various metalloenzymes [2]. Consequently, there is a plethora of adverse clinical signs associated with Cu deficiency in ruminant animals [3]. However, excessive Cu accumulation can also result in clinical toxicity, with adverse outcomes in dairy cattle, including hepatic damage [4], impaired growth performance [5], and mortality due to hemolytic crisis [6]. Evidence suggests that Cu oversupply is a widespread issue within the global dairy industry. For instance, Sinclair and Atkins [7] surveyed the diets of early-lactation cattle across 50 farms in central and northern England, reporting a mean Cu concentration of 27.9 mg/kg of dry matter (DM), which was 2.5 times above the National Research Council (NRC; [8]) recommendations of 11 mg/kg of DM, and 2.8 times above the revised requirements of 10 mg/kg of DM proposed in 2021 [9]. Similarly, Castillo et al. [10] surveyed 39 Californian dairy herds, reporting a mean Cu concentration of 18.0 mg/kg of DM, again exceeding NRC [8] guidelines for the time by a factor of almost 1.9 times [8]. This widespread over-supplementation of adult dairy cattle may contribute to the findings of Kendall et al. [11], who reported that 40% of UK dairy cull cows surveyed had hepatic Cu concentrations above the 508 mg/kg of DM, proposed as the diagnostic threshold for chronic Cu poisoning by Laven and Livesey [12]. This elevated Cu status has resulted in the UK Animal and Plant Health Agency [13] recording 148 confirmed cases of clinical Cu toxicity in UK cattle between 2013 and 2024, of which the majority were from dairy herds. The UK experience is not unique, as Johnston et al. [14] reported chronic Cu toxicity in New Zealand, whilst Bradley [15] documented similar occurrences in an Irish dairy herd.

Subclinical Cu toxicity in cattle has also been identified as a concern, as noted by McCaughern et al. [16] who reported that supplementation at levels below those commonly provided on farms, but exceeding animal requirements, led to a 17.5% reduction in the conception rate of replacement Holstein-Friesian heifers. The boundary at which hepatic Cu concentrations become deleterious was later quantified by Marsh et al. [17] who identified a reduction in conception rates to first and second services when hepatic Cu concentrations exceeded a threshold level of 260 mg/kg of DM. This work is not alone in highlighting potential issues surrounding subclinical Cu toxicity, with a study by Hawkins [18] demonstrating that administering 200 mg of Cu as Ca-Cu-EDTA pre-mating led to decreased 21-day submission and conception rates in New Zealand dairy herds.

While the reproductive consequences of subclinical Cu toxicity in adult cattle are increasingly well documented, far less attention has been paid to the potential risks associated with Cu provision earlier in life. Indeed, the paradox surrounding Cu provision is not confined to adult cattle, whereby appropriate levels of Cu supplementation for artificially reared calves also remain unclear. The European Commission’s Regulation (EU) 2018/1039 sets the legal limit for Cu supplementation in pre-ruminant bovines at 15 mg/kg of complete feed, with a moisture content of 12% (equivalent to 17 mg/kg of DM); however, Hunter et al. [19] suggested that concentrations as low as 10 mg/kg of DM could already pose toxicity risks, noting pre-ruminant calf requirements may be as low as 1 to 2 mg Cu/kg of DM. The NASEM [9] recommends supplementing young calves with 5 mg Cu/kg of DM in milk replacer, and 12 mg/kg of DM in starter and grower feed, despite reporting that the Cu content of whole milk ranges between 0.1 and 1.1 mg/kg of DM. This recommendation for feedstuffs provided to artificially reared calves represents a substantial increase over the concentrations naturally consumed by suckled calves [9]. Indeed, Puschner et al. [20] reported liver Cu concentrations to be 135 to 200 mg/kg of DM higher in artificially reared dairy calves compared to suckled beef calves throughout their first year of life. At the time, the authors attributed this difference to the higher Cu content of 10.0 mg/liter in the milk replacement powder fed to the artificially reared calves, compared to 0.15 mg/liter within the whole milk consumed by suckled beef calves [20]. Similar to adult cattle, reports of Cu toxicity have been reported in calves. For example, Sullivan et al. [21] documented the deaths of seven veal calves aged 10 to 16 weeks from five separate farms in Indiana, USA, due to Cu toxicosis; however, it is important to note that the dietary Cu concentrations associated with these cases were not specified. Additionally, the calves displayed clinical signs similar to those reported by Hunter et al. [19], who described a morbidity rate of 28% among heifer calves on a UK dairy farm. In that longitudinal study, hepatopathy was associated with diets containing 10.1 mg Cu/kg of DM in milk powder and 70.5 mg Cu/kg of DM in creep feed in 2006. Following the removal of supplemental Cu, hepatopathy persisted in 2011, when Cu concentrations in milk replacer were minimal, although creep feed still contained approximately 35 mg Cu/kg of DM derived from natural ingredients. More recently, Henderson et al. [22] reported that Cu supplementation above animal requirements in dairy-beef crossbred calves aged approximately eight weeks did not affect immune function but may have influenced inflammatory pathways, despite no clinical signs of Cu toxicity being observed, concluding that more research into the potential effects of subclinical toxicity is needed in calves.

Calves may be at a particular risk of developing Cu toxicity due to a substantially higher dietary absorption before a functioning rumen is present [12, 23]. For example, Bremner and Davies [23] used re-entrant cannulae to demonstrate that pre-weaned calves can absorb as much as 68% of total dietary Cu across the entire gastrointestinal tract. However, following the anatomical and physiological changes associated with weaning and the onset of rumen function, Cu absorption is markedly reduced, with the authors noting a 40% decline in ileal Cu absorption post-weaning, while overall gastrointestinal uptake fell to 18%. Suttle [24] further reported that adult cattle absorb less than 10% of dietary Cu, likely due to rumen maturation and the activity of ruminal microflora, which enhances the antagonistic effects of sulfur (S) and molybdenum (Mo), thereby reducing copper absorption.

Given the prevalence of Cu over-supplementation within the dairy sector, its potential to cause harm, and the increased absorption in pre-ruminant animals, a deeper understanding of hepatic Cu metabolism in artificially reared calves is required. The primary aim of this study was to better inform supplementation strategies by estimating Cu retention coefficients in artificially reared Holstein-Friesian calves pre- and post-weaning.

## Materials and Methods

This study utilized bio-banked samples and secondary data collected between March and June of 2024 at the Cochno Farm and Research Centre, University of Glasgow, Clydebank, UK. The original study evaluated the effect of two different dietary treatments on rumen development in Holstein-Friesian bull calves. This subsequent study aimed to utilize the tissue samples generated in conjunction with recorded animal performance to determine pre- and post-weaning Cu retention coefficients.

The animal management performed by Yerby et al. [25] can be summarized as follows. Thirty-five Holstein-Friesian bull calves aged 3.8 ± 1.28 weeks (mean ± standard deviation) and weighing 52 ± 18.0 kg were sourced and transported to Cochno Farm and Research Centre. Before commencing the study, five calves were euthanized in accordance with Schedule 1 of the Animals (Scientific Procedures) Act 1986 (approved humane killing methods) and used as a reference group to establish baseline hepatic Cu. The remaining 30 calves were randomly allocated to either a control group (*n* = 10) which received no dietary modifications; a group (*n* = 10) supplemented with 3 g/d of a solid state fermentation product, derived from *Aspergillus niger* (ANP; Synergen™, Alltech, Nicholasville, KY, USA), or a final grouping (*n* = 10) supplemented with 3 g/d of ANP + 2 g/d of a mannan-rich fraction derived from *Saccharomyces cerevisiae* cell walls (MRF™, Alltech, Nicholasville, KY, USA). Dietary treatments were fed for a total period of 9 weeks; pre-weaning treatment administration was via the morning milk feed, whilst treatments were incorporated into the calf concentrate during the post-weaning period.

Calves were housed individually; pre-weaning pens measured 180 × 180 cm; post-weaning, the pens were expanded to 180 × 360 cm. All pens were bedded with barley straw, which was replenished daily and fully cleaned every two weeks. All calves had *ad-libitum* access to water, barley straw, and a commercially available starter calf concentrate (I’Anson Early Weaner Pellets, Yorkshire, UK), which was provided fresh daily and had a declared fresh weight composition of 180 g of crude protein/kg, 87.5 g of crude fiber/kg, 42.5 g of fat/kg, 75 g of ash/kg and a measured Cu concentration of 16.8 mg/kg of DM. In addition, calves were offered 3 L of milk replacer (Volac Heiferlac, Royston, Hertfordshire, UK) twice daily at 07:00 and 16:00. The milk replacement powder had a declared composition of 260 g of crude protein/kg of fresh weight, 160 g of fat/kg of fresh weight, and 19.7 MJ ME/kg of DM with a measured Cu concentration of 2.8 mg/kg of DM. The milk replacement powder was reconstituted according to the manufacturer’s instructions (150 g powder per 850 ml water) and offered at 38 to 40 °C. Calves were weaned abruptly at 6.8 weeks of age, after which they remained on concentrate feed and barley straw only. Throughout the study, calves were reared under conditions that are typical of commercial practice within the UK.

### Data Collection

Calf concentrate refusals were collected and intake recorded daily, whilst weekly samples of milk powder, concentrate, and water were collected, stored at -20 °C, bulked monthly, and sub-sampled for mineral analysis. Calves were also weighed on two consecutive days using digital scales immediately after their morning feed at a weekly interval pre-weaning, extending to a duration of fortnightly post-weaning. Five calves from each treatment (*n* = 15) were euthanized at 6.8 and 12.8 weeks of age in accordance with Schedule 1 of the Animals (Scientific Procedures) Act 1986 (approved humane killing methods). At each point, 10 ml of digesta was collected for volatile fatty acid (VFA) analysis from the ruminal ventral sac and stored at -80 °C following the addition of 0.5 ml metaphosphoric acid. Liver and kidney samples were also collected and stored at − 20 °C for mineral analysis.

Milk powder, calf concentrate, and treatment minerals were extracted and analyzed by inductively coupled plasma-mass spectrometry (ICP-MS; Nexion 2000; Perkin Elmer, Beaconsfield, UK) according to McCaughern et al. [26]. The accuracy of dietary mineral determination was checked via the extraction and analysis of certified European Union (EU) reference hay (BCR-129; Sigma-Aldrich, Dorset, UK) and dairy concentrate samples (BCR-708; Sigma-Aldrich, Dorset, UK), respectively. Additionally, the Cu concentration of liver and kidney samples was determined according to the liver method described by McCaughern et al. [26], with a certified EU reference sample of bovine liver (BCR-185, Sigma-Aldrich, Dorset, UK) being utilized to check analysis accuracy.

Ruminal volatile fatty acids were determined according to Johnson et al. [27] using a gas chromatograph (8860 GC system; Agilent, Stockport, UK) fitted with a CP-Wax 58 FFAP CB column (Agilent, Stockport, UK). The initial column temperature was 60 °C, with nitrogen as the carrier gas at a flow rate of 2 mL/min. The oven temperature was initially ramped to 200 °C over 10 min and held for 3 min, followed by a second ramp over 4 min to 220 °C, which was then held for an additional 3 min. Identification of VFA’s was performed by comparing retention times with standards of acetic acid, *n-*propionic acid, *n*-valeric acid, and butyric acid (Merck, Dorset, UK), with quantification performed using 2-ethylbutyric acid (Merek, Dorset, UK) as an internal standard.

### Calculations and Statistical Analysis

For this study, two distinct developmental phases were defined: pre-weaning and post-weaning. Pre-weaning calves were aged between 3.8 and 6.8 weeks. Weaning was defined by the withdrawal of milk at 6.8 weeks of age, after which calves entered the post-weaning phase. The post-weaning period continued until the end of the study, when calves reached 12.8 weeks of age. Straw intakes were predicted in accordance with NASEM [9], as five per cent of total DM intake, with total DM intake derived from measured intakes of milk and concentrate. Straw mineral content was deduced from the study by Jaakkola et al. [28] for Cu (mean: 4.1, range: 1.5–11 mg/kg of DM) and molybdenum (Mo; mean: 0.31, range: 0.1–2.0 mg/kg of DM), and Givens et al. [29] for sulfur (S; 1.8 ± 1.06 g/kg of DM, mean ± standard deviation). Total liver mass was predicted from calf body weight using a ratio of 2.87% from Lin et al. [30]. Total hepatic Cu content was subsequently calculated by multiplying predicted liver mass on a dry matter basis by the associated Cu concentration. Average daily gain was calculated using the mean body weight derived from two consecutive weights, as the change between consecutive time points divided by the number of days between measurements. Adequate Cu intakes were derived for each calf from NASEM [9] using measured animal body weight and average daily gain. In line with NASEM [9], these values are considered *adequate intakes* rather than requirements, reflecting the committee’s updated quantitative approach.

A hepatic Cu retention coefficient was determined for the phase (pre-weaning or post-weaning) that immediately preceded liver sampling, on an individual calf basis, using the following equation:

$$\begin{array}{lc}\:Hepatic\:copper\:retention\:coefficient\:\left(\%\right)=\\\frac{Hepatic\:Cu\:change\:during\:the\:feeding\:period\:\:\left(\mathrm{m}\mathrm{g}\right)}{Total\:intake\:of\:Cu\:over\:the\:feeding\:period\:\left(\mathrm{m}\mathrm{g}\right)}\:\times\:100\end{array}$$

Revised hepatic steady-state requirements were then calculated for each animal, using the following equation:

$$\begin{array}{lc}\:Hepatic\:steady\: state\:requirements\:for\: phase\:(mg/d)\:=\:\\\:\frac{\left(Total\:dietary\:copper\:intake\:across\:the\:phase\:\right(mg)\:-\:Total\:hepatic\:copper\:change\:across\:the\:phase\:(mg\left)\right)}{Total\:number\:of\:days\:spent\:across\:the\:phase}\end{array}$$

This quantity of dietary Cu represents the amount needed for increased tissue size due to growth and maintenance of metabolic function, whilst assuming no net hepatic Cu concentration change over time.

Statistical analyses were carried out using R (version 4.4.1 [31]) and the following packages used: Dplyr [32], emmeans [33], gremlin [34], Stringr [35] tidyr [36]. Before commencing statistical analyses, a Shapiro–Wilk test was conducted to assess the data distribution for each variable. Restricted maximum likelihood (REML) mixed-effects models were used to assess differences in calf intakes, growth performance, and Cu requirements before and after weaning. The models included the weaning stage (pre- vs. post-weaning), treatment group (control, ANP, or ANP + MRF), and their interaction as fixed effects, with calf ID included as a random effect to account for repeated measurements from the same animal.

In contrast, changes in liver Cu concentrations, kidney Cu concentrations, and VFAs at each sampling point were analyzed using linear mixed-effects models. Where possible, models included weaning stage (pre- vs. post-weaning), treatment group, and their interaction as fixed effects. For variables where no treatment was applied at the initial sampling point, models included weaning stage and treatment group as fixed effects without their interaction. Statistical significance was declared at *P* < 0.05. Results pertaining to the weaning phase are presented as the predicted means and their associated standard error of difference (SED).

## Results

Mean concentrate intake increased markedly across the weaning period (F = 826.43, *P* < 0.0001), rising from 348 to 2,938 g of DM/d (Table 1). This pattern was reflected in total dry matter intake, which also increased from 1,241 to 3,049 g of DM/d (F = 408.60, *P* < 0.0001). Calves grew throughout the study with a mean initial liveweight of 57.3 kg, which increased (F = 277.65, *P* < 0.0001) to 73.8 kg at weaning, before a further increase (F = 378.02, *P* < 0.0001) to 124.2 kg at the end of the study. Mean daily liveweight gain increased (F = 31.56, *P* = 0.0006) from 0.87 kg/d pre-weaning to 1.22 kg/d post-weaning. Total intake of Cu, S, and Mo all increased following weaning, with mean intakes rising from 8.2 to 49.3 mg/d for Cu (F = 738.72, *P <* 0.0001), 7.87 to 13.08 g/d for S (F = 168.87, *P <* 0.0001), and 0.93 to 3.05 mg/d for Mo (F = 515.77, *P <* 0.0001), respectively. This corresponded with significant changes in dietary mineral concentrations, where Cu concentration increased from 6.4 to 16.1 mg/kg of DM (F = 604.96, *P* < 0.0001), Mo concentration increased from 0.74 to 1.00 mg/kg of DM (F = 560.81, *P* < 0.0001), and S concentration decreased from 6.37 to 4.26 g/kg of DM (F = 770.95, *P* < 0.0001). The estimated intake of barley straw bedding contributed comparatively small additional quantities of these minerals. Based on reported ranges for barley straw mineral composition, estimated Cu intake from straw ranged from 0.1 to 0.6 mg/d pre-weaning and from 0.2 to 1.4 mg/d post-weaning, assuming mean straw dry matter intakes of 50 and 123 g/d, respectively. Corresponding estimated contributions from straw ranged from 0.01 to 0.10 mg/d pre-weaning and 0.01 to 0.25 mg/d post-weaning for Mo, and from 0.04 to 0.14 g/d pre-weaning and 0.09 to 0.35 g/d post-weaning for S.

Mean hepatic Cu concentrations did not differ (F = 0.86, *P =* 0.3655) over the pre-weaning period, but decreased (F = 7.22, *P* = 0.0197) from 516 mg/kg of DM to 441 mg/kg of DM during the post-weaning period. Considerable inter-animal variation was observed, with hepatic Cu concentrations ranging from 311 to 675 mg/kg of DM throughout the study. Kidney Cu concentration was also not affected by weaning (F = 0.89, *P* > 0.05) and ranged from 10.9 to 44.6 mg/kg of DM throughout the study. There was no effect of treatment from the original study on Cu intake or status (*P >* 0.05).

Mean ruminal acetate concentrations increased (F = 6.31, *P* = 0.0224) during the pre-weaning period, rising from 54.8 to 73.4 mM, before stabilizing (F = 3.27, *P =* 0.0835) during the post-weaning phase. Ruminal propionate concentrations also increased (F = 12.97, *P* = 0.0022) during the pre-weaning period (22.2 vs. 30.5 mM), before further increasing (F = 19.71, *P* = 0.0002) during the post-weaning phase to 47.3 mM. Ruminal butyrate concentrations increased (F = 22.96, *P* = 0.0002) during the pre-weaning stage from 7.1 to 17.3 mM, and continued to increase (F = 6.34, *P* = 0.0124) during the post-weaning period to 22.1 mM by the end of the study. Mean ruminal valerate concentrations increased during the pre-weaning period (F = 9.62, *P* = 0.0065) from 1.4 to 3.0 mM before further increasing (F = 37.23, *P* < 0.0001) during the post-weaning phase from 3.0 to 6.7 mM. Overall, total VFA concentrations increased (F = 14.60, *P =* 0.0028) from 85.5 to 124.1 mM over the pre-weaning phase, with a further increase (F = 12.46, *P* = 0.0022) to 162.0 mM by the end of the study.

**Table 1:** Performance, volatile fatty acid profile, and copper status of Holstein-Friesian bull calves pre- and post- weaning

		Phase^a^				
Explanatory parameter^b^		Pre-weaning	Post-weaning	SED	F-statistic	*P*-value
Intake						
Milk powder intake, g of DM/d		841	0	--	--	--
Concentrate intake, g of DM/d		348	2,938	92.3	826.43	< 0.0001
Straw intake, g of DM/d^c^		50	123	--	--	--
Total intake, g of DM/d		1,241	3,049	90.1	408.60	< 0.0001
Growth performance						
Initial liveweight, kg		57.3	73.8	0.94	277.65	< 0.0001
Final liveweight, kg		73.8	124.2	2.63	378.02	< 0.0001
Liveweight change, kg/d		0.87	1.22	0.064	31.56	0.0006
Mineral intake^d^						
Copper intake, mg/d		8.2	49.3	1.55	738.72	< 0.0001
Sulfur intake, g/d		7.87	13.08	0.411	168.87	< 0.0001
Molybdenum intake, mg/d		0.93	3.05	0.096	515.77	< 0.0001
Copper concentration, mg/kg of DM		6.4	16.1	0.41	604.96	< 0.0001
Sulfur concentration, g/kg of DM		6.37	4.26	0.078	770.95	< 0.0001
Molybdenum concentration, mg/kg of DM		0.74	1.00	0.011	560.81	< 0.0001
Hepatic copper^e^						
Initial concentration, mg/kg of DM		469 (312–675)	516 (433–664)	66.2	0.86	0.3655
Final concentration, mg/kg of DM		516 (433–664)	441 (311–622)	28.0	7.22	0.0197
Kidney copper^e^						
Initial concentration, mg/kg of DM		31.7 (27.5–35.5)	26.1 (10.9–44.5)	4.40	1.59	0.2249
Final concentration, mg/kg of DM		26.1 (10.9–44.5)	25.4 (13.8–44.6)	3.77	0.04	0.8549
Ruminal volatile fatty acids						
Initial acetate, mM		54.8	73.4	9.40	6.31	0.0224
Final acetate, mM		73.4	86.3	7.17	3.27	0.0835
Initial propionate, mM		22.2	30.5	3.52	12.97	0.0022
Final propionate, mM		30.5	47.3	3.80	19.71	0.0002
Initial butyrate, mM		7.1	17.3	3.19	22.96	0.0002
Final butyrate, mM		17.3	22.1	1.96	6.34	0.0124
Initial valerate, mM		1.4	3.0	0.72	9.62	0.0065
Final valerate, mM		3.0	6.7	0.61	37.23	< 0.0001
Initial total, mM		85.5	124.1	15.60	14.60	0.0028
Final total, mM		124.1	162.0	10.90	12.46	0.0022

^a^ Pre-weaning calves were between 3.8 and 6.8 weeks of age; post-weaning calves were 6.8 to 12.8 weeks of age

^b^ Restricted maximum likelihood (REML) mixed-effects models were used to assess intake, growth performance, and mineral intake, with weaning phase * treatment group, and their interaction as fixed effects, and calf ID as a random effect. Hepatic Cu, kidney Cu, and ruminal VFAs were analyzed using linear mixed-effects models, where possible models included weaning stage * treatment group, and their interaction as fixed effects. For variables where no treatment was applied at the initial sampling point, models included weaning stage and treatment group as fixed effects

^c^ Predicted using NASEM [9]^d^ Straw minerals predicted using Jaakkola et al. [28] for copper and sulfur, and Givens et al. [29] for molybdenum

^e^ Range presented in parentheses

Derived total hepatic Cu increased over the course of the study; pre-weaning, the total amount rose (F = 9.20, *P* = 0.0076) from 212 to 284 mg, rising further post-weaning (F = 15.19, *P* = 0.0007) to 399 mg (Table 2). Weaning affected (F = 13.40, *P* = 0.0015) hepatic Cu change, with a net increase of 2.5 mg/kg of DM/day pre-weaning, in contrast to a net decrease of 1.8 mg/kg of DM/day post-weaning.

Copper adequate intake, determined from measured values according to NASEM [9], increased (F = 600.98, *P* < 0.0001) from 6.2 mg/day (5.4 mg/kg of DM) during the pre-weaning phase to 46.4 mg/day (14.9 mg/kg of DM) post-weaning. In contrast, the estimated coefficient of hepatic Cu retention declined (F = 208.03, *P* < 0.0001) from a mean of 81% to 4.9% following weaning. Consequently, hepatic steady-state Cu requirements increased (F = 267.62, *P* < 0.0001) from 4.2 mg/day (3.2 mg/kg of DM) pre-weaning to 46.4 mg/day (15.2 mg/kg of DM) post-weaning.

**Table 2:** Derived pre- and post-weaning differences in hepatic copper status and copper balance in Holstein–Friesian bull calves

		Phase^a^				
Explanatory parameter^b^		Pre-weaning	Post-weaning	SED	F-statistic	*P*-value
Hepatic copper^c^						
Total initial, mg		212 (126–344)	284 (226–359)	36.3	9.20	0.0076
Total final, mg		284 (226–359)	399 (238–586)	29.5	15.19	0.0007
Total Cu change, mg		114.7	86.5	29.50	0.92	0.3475
Hepatic Cu change, mg/kg of DM/d		2.5	-1.8	1.18	13.40	0.0015
Copper balance						
Copper adequate intake, mg/d^d^		6.2	46.4	1.60	600.98	< 0.0001
Copper adequate intake, mg/kg of DM^d^		5.4	14.9	0.26	1659.23	< 0.0001
Hepatic copper retention coefficient, %		81.1	4.9	5.33	208.83	< 0.0001
Hepatic steady-state requirements mg/d		4.2	46.4	2.59	267.62	< 0.0001
Hepatic steady-state requirements mg/kg of DM		3.2	15.2	0.63	368.60	< 0.0001

^a^ Pre-weaning calves were between 3.8 and 6.8 weeks of age; post-weaning calves were 6.8 to 12.8 weeks of age

^b^ Results were analyzed using linear mixed-effects models where possible models included weaning stage * treatment group, and their interaction as fixed effects. For variables where no treatment was applied at the initial sampling point, models included weaning stage and treatment group as fixed effects

^c^ Range presented in parentheses

^d^ Calculated using animal performance data in line with NASEM [9]

## Discussion

There is growing evidence to suggest harmful subclinical implications of an increased hepatic Cu status without the demonstration of clinical Cu poisoning, as exemplified by reduced conception rates, altered liver function in dairy heifers [16], and shifted inflammation pathways [22]. Artificially reared dairy calves may be particularly vulnerable due to the high concentration of Cu in milk replacer and calf concentrate, both provided during the pre-ruminant stage [19, 20]. Studies have demonstrated that pre-ruminant calves absorb Cu more efficiently than mature ruminants, resulting in rapid hepatic accumulation during early life [20]. As the liver is the primary Cu storage site in cattle, excessive accumulation during early development may increase the risk of adverse effects due to clinical or sub-clinical toxicity [19, 37].

### Calf Intakes and Growth Performance

Calves utilized in this study were managed under conditions representative of commercial dairy production systems in the UK, France, and North America [38–40]. Calves were fed restricted milk allowances of three liters twice daily, which aligns with common UK management practices. Indeed, 87.5% of UK farms feed calves twice daily, with 51% of these farms providing between four and seven liters of milk per day [38] such feeding regimes are commonly adopted to comply with legislative requirements, including the Welfare of Farmed Animals (England) Regulations 2007 and European Council Directive 2008/119/EC on minimum standards for the protection of calves, both of which require calves be fed at least twice daily. Whilst the twice-a-day feeding approach is standard practice in the UK, it is also used on approximately 25% of French farms [40] and was reported on 114 out of the 115 Canadian farms surveyed by Vasseur et al. [39], where calves also received 5.5 L of milk per day across two feeds. In addition to restricted milk feeding, *ad-libitum* access to both concentrate feed and forage reflects common industry practice. It is a calf feeding strategy that is used by 52.8% of UK farmers [38], 91% of Brazilian farmers [41] and 65.5% of Canadian farmers [39]. However, forage was introduced to the calves used in our current study earlier than the US average introductory age of 24.5 days [42], with intakes of both feedstuffs being permitted prior to the start of the study. Calves were also fed a commercially available milk replacer that was mixed at concentrations typical of UK management systems [38].

The use of industry-representative feeding practices resulted in growth rates comparable with those reported in commercial dairy systems. Bazeley et al. [43] reported a median growth rate of 0.12 kg/d in 319 Holstein calves aged 8–30 days in the UK, while Archer [44] reported a mean daily gain of 0.77 kg/d in calves up to six months of age. Comparable growth rates have also been reported internationally. Tautenhahn et al. [45] observed median daily liveweight gains of 0.68 kg/d across 50 German dairy farms, while Van De Stroet et al. [46] reported gains of 0.64 kg/d in 281 Holstein heifer calves from six studies conducted in Pennsylvania, USA, with both authors noting higher growth rates in younger animals, which slowed after weaning.

### Hepatic Copper Concentrations

Despite calves in the present study being reared under conditions typical of industry practice, their Cu intakes exceeded published nutritional recommendations. Historically, the NRC [8] recommended a Cu concentration of 10 mg/kg of DM for milk replacer, starter, and grower concentrate feeds for young calves, despite acknowledging that whole milk contains substantially lower concentrations of Cu (0.1–1.1 mg/kg of DM). However, NASEM [9] revised these recommendations in 2021, replacing animal requirements with adequate intake values. In this revision, the recommended Cu concentration for milk replacers was reduced to 5 mg/kg of DM, while adequate intakes for starter and grower concentrate feeds were increased to 12 mg/kg of DM. In addition, NASEM [9] proposed an equation to estimate individual adequate Cu intakes based on body weight and average daily liveweight gain. Using these calculations, calves in our study exceeded the predicted adequate Cu intake by 121% (+ 2.3 mg/d) during the pre-weaning phase, and 109% (+ 3.8 mg/d) during the post-weaning phase. However, it is important to consider these intakes in the context of regulatory limits. Under European Commission Implementing Regulation (EU) 2018/1039, the maximum permitted Cu concentration in complete feed for calves before rumination is 15 mg/kg of fresh matter (approximately 17 mg/kg of DM), whilst the upper limit for older cattle is 30 mg/kg of fresh matter (approximately 34 mg/kg of DM). The total dietary Cu concentrations provided to calves in our study remained well below these regulatory limits. Similarly, the Canadian Food Inspection Agency permits up to 40 mg Cu/kg of DM in cattle feeds, including calf feeds [47], meaning that Cu concentrations used in the present study would also fall within permitted limits in other feeding systems. Furthermore, when Cu requirements were calculated using hepatic steady-state estimates derived from our study, calves were receiving approximately 168% (+ 4.2 mg/d) of their calculated requirement during the pre-weaning phase, and 106% (+ 2.7 g/d) during the post-weaning phase.

This overfeeding of Cu may have contributed to the consistently high hepatic Cu concentrations observed in our study, with all calves exceeding 260 mg/kg of DM, a threshold previously associated with optimal fertility, and 322 mg/kg of DM, which has been linked to a decline in liver health [17]. Furthermore, 40% of calves exceeded the veterinary diagnostic threshold for Cu toxicity (508 mg/kg of DM; [12]) at 3.8 weeks of age. Similar findings have been reported by McCaughern et al. [16], who observed mean hepatic Cu concentrations of 706 mg/kg of DM in heifers aged 6.9 months that had also been reared under conditions representative of UK commercial dairy systems. Although the hepatic Cu concentrations of calves at birth were unknown, maternal transfer of Cu to the developing fetus has previously been reported [48]. When considered alongside the excessive Cu supplementation that routinely occurs in adult dairy cattle [7], it is plausible that calves were born with already elevated hepatic Cu concentrations. However, previous studies have not identified a correlation between liver Cu concentrations in dams and their offspring [49, 50]. Nevertheless, the applicability of these studies to modern dairy systems may be limited, as the hepatic Cu concentrations of dams were substantially lower than those reported in contemporary lactating Holstein-Friesian dairy cows by McCaughern et al. [26] and Kendall et al. [11].

Regardless of whether these elevated hepatic Cu concentrations were present at birth or developed during the weeks preceding enrolment, dietary Cu provision during the study was sufficient to maintain hepatic Cu concentrations at levels associated with liver damage in Holstein-Friesian heifers [17]. This persistence of elevated hepatic Cu concentrations is concerning, as Cu management during early life may have important implications for lifelong health and productivity. Excess hepatic Cu accumulated during the pre-weaning period may persist into later stages of development, particularly given the limited capacity of ruminants to excrete Cu [51]. If elevated hepatic Cu concentrations persist until the average age at first service (15.8 months; [52]), reproductive performance could potentially be influenced. Indeed, McCaughern et al. [16] reported a 17.5% reduction in the fertility of heifers at increased hepatic Cu concentrations, suggesting a possible association between elevated hepatic Cu stores and reduced reproductive performance. This sensitivity of reproductive physiology to Cu status is further supported by Hawkins [18], who demonstrated that administering 200 mg of Cu as Ca-Cu-EDTA 10 days prior to the pre-service mating period in New Zealand dairy herds was associated with reductions in both 21-day submission and conception rates. The implications of impaired fertility may extend beyond reproduction alone, with downstream effects on overall productivity. Macrae and Esslemont [53] demonstrated that a delayed first-calving past 26 months of age is associated with reduced lifetime milk yield and fewer productive lactations, highlighting the potential long-term economic and biological consequences of excessive Cu accumulation during early life.

The findings of the present study align with those of McCaughern et al. [16] who attributed elevated hepatic Cu concentrations primarily to concentrate intake. The mean total pre-weaning Cu intake from milk replacer was 45 mg, whereas Cu intake from concentrate alone was 110 mg, indicating that concentrates were the predominant source of dietary Cu. A comparable role of concentrate intake in hepatic Cu accumulation was proposed by Puschner et al. [20], who reported that artificially reared dairy calves exhibited hepatic Cu concentrations approximately 50–60 mg/kg of fresh weight higher than those of suckler beef calves. This discrepancy was attributed to differences in feeding management, particularly the earlier introduction of concentrate feeds in dairy systems. Indeed, the European Food Safety Authority [54] noted that concentrates are typically introduced approximately four weeks before weaning in beef cattle, with weaning occurring between 5 and 11 months of age; however, in dairy production systems, calves may be exposed to concentrates from as early as birth, potentially increasing Cu intake during periods of high absorptive capacity. Although it is important to note that the milk replacer powder used in this study contained 2.8 mg Cu/kg of DM, which is only 56% of the adequate intake value proposed by NASEM [9], however, this concentration is still 2.6-fold greater than the maximum Cu concentration reported in whole milk [8] and therefore should not be overlooked as a potential contributor to elevated hepatic Cu concentrations in young calves.

In contrast to hepatic Cu concentrations, kidney Cu concentrations in our current study remained within normal physiological limits (< 55 mg/kg of DM) for all animals [6]. These findings may suggest that pre-ruminant calves possess a greater capacity to tolerate elevated Cu intakes than adult cattle, likely due to the liver’s ability to sequester excess Cu safely during early life. Bozhkov et al. [55] proposed that such tolerance may be related to differences in metallothionein production and utilization across mammalian species, which influence the ability of hepatic tissues to bind and store excess Cu. Nevertheless, prolonged oversupply of dietary Cu may eventually overwhelm hepatic storage mechanisms, potentially leading to toxicity and mortality, as previously reported in calves by Hunter et al. [19].

One potential factor contributing to the elevated Cu stores observed in the present study is the continued reliance on outdated assumptions regarding Cu absorption efficiency. Many absorption coefficients currently used in requirement calculations have historically been extrapolated from studies conducted in lambs [23]. Within the requirement prediction models proposed by NASEM [9], a pre-weaning Cu absorption coefficient of 50% is applied, which assumes the calf is consuming both milk replacer and starter feed. In contrast, Suttle [37] reported considerably higher Cu absorption in solely milk-fed lambs, measuring 71 ± 3.7% and 75 ± 5.4% across two experiments, where Cu sulfate pentahydrate was the supplemental source. However, a coefficient of 50% was observed by Bremner and Dalgarno [56] in calves up to 14 weeks of age. Although utilizing animals beyond 12 weeks of age may have underestimated Cu absorption coefficients with respect to current pre-weaning animals, as these would now typically be classified as post-weaning under modern UK [38], French [57], and Canadian [39] production systems. Nevertheless, this age range likely reflected prevailing weaning practices at the time the study was conducted [58], emphasizing the need for updated research as calf management practices continue to evolve.

Despite being derived from lambs, the absorption value of 75% reported by Suttle [37] aligns broadly with the 81% pre-weaning estimated retention value obtained in our current study. Furthermore, in the present study, Cu retention decreased to 5 ± 5.0% post-weaning, closely matching the post-weaning absorption of 8.3 ± 3.4% reported by Suttle [37]. A similar pattern was also noted by Bremner and Davies [23] who reported a Cu absorption coefficient in artificially reared calves of 68% pre-weaning, which decreased to 27% post-weaning. Collectively, these findings indicate that Cu absorption in calves is highly dynamic and strongly influenced by developmental stage. Consequently, the application of a single absorption coefficient across both the pre- and post-weaning periods is unlikely to reflect the biological reality of early-life Cu metabolism. Such simplification may lead to systematic overestimation of dietary Cu requirements and increase the risk of inadvertent Cu over-supplementation in modern calf-rearing systems.

### Rumen Development and Copper Retention

The pattern of hepatic Cu concentrations observed in the present study mirrors that reported by Puaschner et al. [20], where hepatic Cu concentrations initially increased before subsequently declining with age. This pattern may reflect the progressive development of the rumen and its influence on Cu absorption. Evidence of rumen maturation was observed in the present study through increasing total VFA production and shifts in ruminal VFA composition. Specifically, acetate decreased as a proportion of total VFAs, while butyrate and valerate increased with age, patterns widely recognized as indicators of advancing rumen development [59, 60]. These changes were accompanied by substantial increases in propionate, butyrate, and valerate concentrations, further supporting the transition from a pre-ruminant to a functional ruminant digestive system [61].

As rumen development progresses, the microbial environment increasingly facilitates interactions between Cu and dietary antagonists such as S and Mo [62], both of which can reduce Cu bioavailability either in isolation or in combination [63]. Consequently, even if dietary Cu intake remains constant or increases, the proportion of Cu absorbed systemically declines as ruminal fermentation becomes established. This mechanism is reflected in the present study by the marked reduction in the estimated hepatic Cu retention coefficient, which decreased from 81% during the pre-weaning phase to 5% post-weaning. Such a decline is consistent with the transition from the highly efficient pre-weaning absorption observed in milk-fed calves to the lower Cu availability typical of mature ruminant digestion [37].

The rate of rumen development is influenced by several management factors, particularly feeding strategy. Yohe et al. [64] and Coverdale [65] demonstrated that concentrate feed intake plays a critical role in stimulating rumen epithelial development and fermentation capacity. When calves are offered concentrate feed *ad-libitum*, individual variation in intake can result in differing rates of rumen maturation [66]. This variability may explain the greater variation in hepatic Cu concentrations observed at 12.8 weeks of age compared with 6.8 weeks in the present study, as calves with more advanced rumen development would likely experience greater ruminal Cu–S–Mo interactions and reduced Cu absorption.

### Implications for Copper Feeding Strategies in Calves

Findings from this study demonstrate a need to reassess the mineral composition of calf diets. The adequate intake proposed by NASEM [9] of 5 mg Cu/kg of DM for milk replacer appears excessive, being approximately 1.5 times higher than the 3 mg/kg of DM suggested by this study for pre-ruminant calves across all feedstuffs. However, it is important to note that all calves included in the study had elevated hepatic Cu concentrations and were therefore likely upregulating Cu excretion beyond normal physiological conditions [67] or downregulating absorptive capacity [68], raising the possibility that even our current estimates of Cu requirements might be inflated, highlighting the need for further investigation under lower dietary Cu concentrations. Indeed, Cu requirements may be considerably lower, as concentrations naturally present in whole milk typically range from 0.09 to 1.79 mg/kg of DM [8]; however, it needs to be considered that a nursing calf will typically consume substantially more milk than a restricted-fed calf. As such, future studies should therefore evaluate calf performance, health, and Cu metabolism across a gradient of lower Cu intakes to better define minimum physiological requirements.

In contrast, post-weaning recommendations remain substantially higher. While this study suggests a requirement of 15 mg/kg of DM, this value is similarly derived from calves with a high Cu status and should therefore be interpreted with caution. Accordingly, additional research is warranted to determine whether comparable retention coefficients and Cu requirements are maintained at lower hepatic Cu concentrations. Although a more appropriate post-weaning value may lie between the lower limit of 12 mg/kg of DM, as proposed by NASEM [9], and the upper estimate of 15 mg/kg of DM identified in our study.

## Conclusion

Copper retention in Holstein–Friesian calves was strongly associated with developmental stage, showing substantially greater retention occurring during the pre-weaning period compared with post-weaning. Pre-weaning calves exhibited an estimated hepatic Cu retention coefficient of 81%, suggesting that these animals may absorb a larger proportion of dietary Cu than mature ruminants. Following weaning, this coefficient declined sharply to 5%, coinciding with advancing rumen development signaled by increasing ruminal fermentation activity and VFA production, driving antagonism of Cu from S and Mo, thereby reducing Cu bioavailability. Consequently, the pre-weaning period may represent a phase of elevated Cu retention and a higher potential for rapid hepatic Cu accumulation.

The current Cu feeding recommendations may not fully account for the high absorptive capacity of pre-ruminant calves. Although dietary Cu concentrations were within commonly recommended limits, calves accumulated substantial hepatic Cu stores during early life. Revised hepatic steady-state requirements indicated that 3 mg Cu/kg of DM may be sufficient to meet requirements for growth and maintenance in calves pre-weaning, which increased to 15 mg/kg of DM post-weaning. In addition, refining Cu recommendations to reflect developmental stage and rumen maturation may help reduce excessive hepatic Cu accumulation while maintaining adequate mineral nutrition. There is a need to evaluate Cu availability at lower hepatic concentrations in pre-ruminant calves, an area which warrants further investigation.

## Data Availability

The data that supports the findings of this study are available from the corresponding author upon reasonable request.
